# Prescribing Antimicrobial Drugs for Acute Gastroenteritis, Primary Care, Australia, 2013–2018

**DOI:** 10.3201/eid2705.203692

**Published:** 2021-05

**Authors:** Wen-Qiang He, Martyn D. Kirk, John Hall, Bette Liu

**Affiliations:** University of New South Wales, Sydney, New South Wales, Australia (W.-Q. He, H. Hall, B. Liu);; Australian National University, Canberra, Australian Capital Territory, Australia (M.D. Kirk)

**Keywords:** acute gastroenteritis, antibiotic prescription, *Campylobacter*, nontyphoidal *Salmonella*, bacteria, antimicrobial resistance, Australia

## Abstract

During 2013–2018, antimicrobial drugs were prescribed for 6.8% of cases of acute gastroenteritis encountered in general practice in Australia, including 35.7% of *Salmonella* infections and 54.1% of *Campylobacter* infections. During that time, prescriptions for acute gastroenteritis decreased by 2.0%. Managing infectious gastroenteritis in general practice will require greater antimicrobial stewardship.

## Abstract 2

Prescriptions for 6.8% of cases suggests a need for greater antimicrobial stewardship.

Worldwide every year, acute gastroenteritis causes a loss of ≈89.5 million disability-adjusted life-years and 1.45 million deaths (*1*). In 2010, an estimated 16.6 million persons in Australia (population 22 million [*2*]) were affected, and ≈1.1 million of these persons sought care at a general practice (*3*,*4*). The most common cause of acute gastroenteritis is viral infection; therefore, antimicrobial drugs are not routinely recommended (*5*–*7*). Even for some common bacterial causes of acute gastroenteritis (e.g., nontyphoidal *Salmonella* and *Campylobacter* infections), antimicrobial therapy is not required for most patients because these infections are usually self-limiting (*8*).

Overuse of antimicrobial drugs for treating upper respiratory tract infections (mostly caused by viruses) has been well described (*9*,*10*) but not as much for acute gastroenteritis (*11*). Knowing the extent and pattern of antimicrobial drug use for acute gastroenteritis can help determine whether interventions to improve antimicrobial drug use for this specific clinical scenario are warranted. 

We examined prescription of antimicrobial drugs for acute gastroenteritis in primary care practice in Australia during 2013–2018. The study was approved by the MedicineInsight Independent External Data Governance Committee (reference no. 2019-030: December 23, 2019) and the University of New South Wales Human Research Ethics Committee (no. HC190886).

## The Study

We extracted clinical encounters for cases (including multiple episodes/patient) of acute gastroenteritis, nontyphoidal *Salmonella* infection, and *Campylobacter* infection recorded by MedicineInsight, a national primary healthcare database in Australia (https://www.nps.org.au/medicine-insight) during 2013–2018 and examined whether an antimicrobial drug was prescribed on the day of diagnosis (Appendix). Antimicrobial drugs were prescribed for 6.8% (6,652/98,496) of cases of acute gastroenteritis, including 35.7% (391/1,096) cases of nontyphoidal *Salmonella* infection and 54.1% (1,066/1,969) cases of *Campylobacter* infection.

Antimicrobial drug prescriptions for acute gastroenteritis increased with patient age (<10 years, 3.8%; >65 years, 13.7%) (Table 1). Antimicrobial drugs were more likely to be prescribed for those with than without the following: fever or no temperature measurement, a requested fecal sample test, underlying conditions, or a record of bacterial or parasitic infection. Antimicrobial drugs were less likely to be prescribed for those with a record of viral infection. Prescribing also differed by practice remoteness; prescribing was higher in practices in more remote areas than in cities. During the study period, the trend toward antimicrobial drug prescribing decreased from 7.8% to 5.8% (p<0.001). Similar findings were observed for children <10 years of age (Appendix Table 1).

**Table 1:** Proportion of cases of acute gastroenteritis for which antimicrobial drugs were prescribed overall and according to various characteristics, Australia, 2013–2018

Characteristic	No. prescriptions/no. cases (%)	Adjusted odds ratio (95% CI)	p value*
Overall	6,652/98,496 (6.8)		
Age, y			
<10	762/20,130 (3.8)	Referent	
10–29	1,774/30,695 (5.8)	1.56 (1.42–1.71)	<0.001
30–49	2,065/29,315 (7.0)	1.87 (1.71–2.05)	<0.001
50–64	1,093/11,369 (9.6)	2.46 (2.21–2.73)	<0.001
>65	958/6,987 (13.7)	3.27 (2.88–3.71)	<0.001
Sex			
M	3,098/47,892 (6.5)	Referent	
F	3,554/50,604 (7.0)	1.02 (0.97–1.08)	0.41
Aboriginal or Torres Strait Islander			
No	5,076/74,978 (6.8)	Referent	
Yes	145/2,516 (5.8)	0.98 (0.82–1.17)	0.82
Unknown	1,431/21,002 (6.8)		
Concession card holder			
No	3,447/56,841 (6.1)	Referent	
Yes	1,820/22,177 (8.2)	1.04 (0.97–1.12)	0.31
Unknown	1,385/19,478 (7.1)		
Fever, temperature >38.5°C			
No	1,748/30,312 (5.8)	Referent	
Yes	71/566 (12.5)	2.75 (2.09–3.60)	<0.001
Not recorded	4,833/67,618 (7.1)	1.14 (1.07–1.21)	<0.001
Fecal sample test requested			
No	4,832/86,085 (5.6)	Referent	
Yes	1,820/12,411 (14.7)	2.75 (2.58–2.92)	<0.001
Etiology			
Not recorded	5,820/79,799 (7.3)	Referent	
Viral	342/17,896 (1.9)	0.30 (0.27–0.34)	<0.001
Bacterial	483/790 (61.1)	19.49 (16.66–22.80)	<0.001
Parasitic	7/11 (63.6)	24.12 (6.22–93.59)	<0.001
Underlying conditions†			
No	5,314/85,970 (6.2)	Referent	
Yes	1,338/12,526 (10.7)	1.09 (1.00–1.19)	0.04
No visits to general practitioner in past year			
0–7	5,000/74,630 (6.7)	Referent	
8–14	950/14,332 (6.6)	1.02 (0.94–1.10)	0.60
>15	702/9,534 (7.4)	0.95 (0.86–1.04)	0.28
Remoteness of practice			
Major city	4,421/69,557 (6.4)	Referent	
Inner regional	1,172/16,438 (7.1)	0.97 (0.90–1.04)	0.35
Outer regional or remote	1,059/12,501 (8.5)	1.21 (1.12–1.30)	<0.001
Year of diagnosis			
2013	1,238/15,845 (7.8)	Referent	
2014	1,258/16,681 (7.5)	0.92 (0.84–1.00)	0.046
2015	1,165/16,912 (6.9)	0.84 (0.77–0.92)	<0.001
2016	1,143/17,613 (6.5)	0.77 (0.71–0.84)	<0.001
2017	1,008/16,995 (5.9)	0.71 (0.65–0.78)	<0.001
2018	840/14,450 (5.8)	0.71 (0.65–0.78)	<0.001
*Adjusted for all variables listed in the table. †Any medical history of diabetes mellitus, arthritis, or chronic kidney disease.			

**Figure 1:**
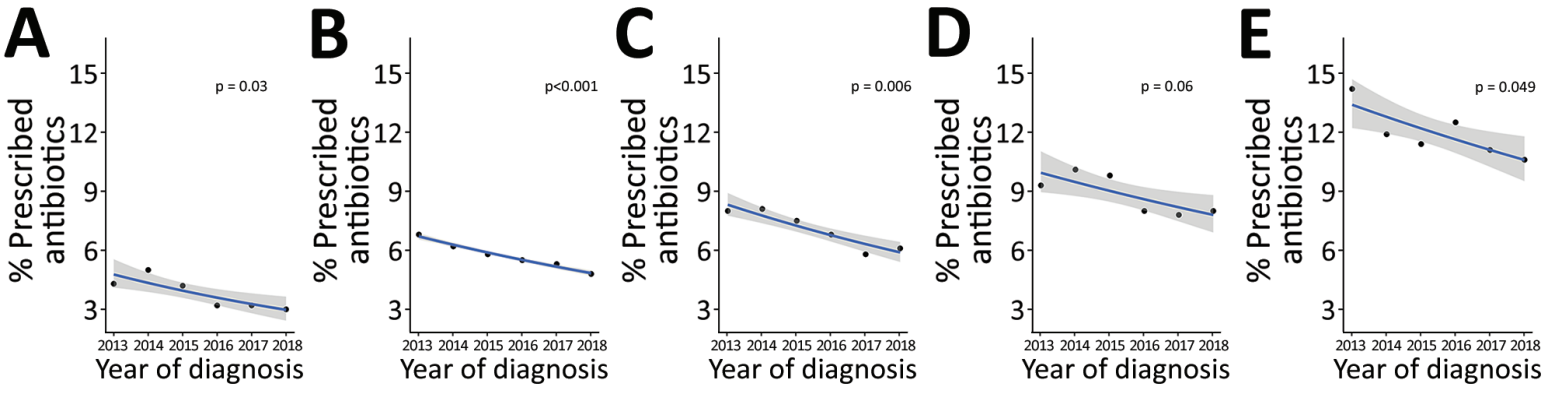
Proportion of acute gastroenteritis cases for which antimicrobial drugs were prescribed, by year of diagnosis and patient age, Australia, 2013–2018. A) <10 y; B) 10–29 y; C) 30–49 y; D) 50-64 y; E) >65 y.

The greatest reductions in antimicrobial drug prescriptions were found for those >65 years of age (2.8% absolute reduction from 13.4% to 10.6% (p = 0.049). The next greatest reductions were for those 30–49 years of age (2.4% absolute reduction from 8.3% to 5.9%; p = 0.006), 10–29 years (from 6.7% to 4.8%; p<0.001), and <10 years (from 4.8% to 3.0%; p = 0.03) (Figure 1).

For patients with nontyphoidal *Salmonella* infection (Appendix Table 2), prescriptions for antimicrobial drugs were more likely for those 30–49 than those <10 years of age (41.7% vs. 34.1%; p = 0.02) and in practices in outer regional or remote areas than in cities. Trend analysis of antimicrobial drug prescriptions for patients with nontyphoidal *Salmonella* infection suggested a significant reduction; absolute reduction was 11.4% (from 42.1% in 2013 to 30.7% in 2018; p = 0.01). For patients with *Campylobacter* infection (Appendix Table 3), antimicrobial drugs were more likely to be prescribed for female than male patients (56.8% vs. 51.7%; p = 0.02). We observed no significant reduction in antimicrobial drug prescriptions for patients with *Campylobacter* infection (55.8% to 57.1%; p = 0.81).

Of the 6,652 acute gastroenteritis cases for which antimicrobial drugs were prescribed, a reason was recorded for 42.9% (2,854/6,652), including 80.4% (2,295/2,854) for acute gastroenteritis, 1.1% (30/2,854) for other gastrointestinal illnesses, 5.7% (162/2,854) for respiratory tract infections, 1.8% (50/2,854) for urinary tract infections, and 11.1% (317/2,854) for other reasons. Of the 6,652 acute gastroenteritis cases for which antimicrobial drugs were prescribed, 7,159 prescriptions were written: 1 for 92.9% (6,179/6,652) of cases and >2 (range 2–5) for 7.1% (473/6,652). The predominant class of drug prescribed for acute gastroenteritis was nitroimidazoles (41.6% of total; Table 2), of which metronidazole accounted for the most prescriptions (24.7% of total; Appendix Table 4).

**Table 2:** Classes of antimicrobial drugs prescribed for cases of acute gastroenteritis, nontyphoidal *Salmonella* infection, and *Campylobacter* infection, Australia, 2013–2018

Case type, drug class	No. prescriptions	Proportion of total prescriptions, %
Acute gastroenteritis, 7,159 cases		
Nitroimidazoles	2980	41.6
Quinolones	1059	14.8
Penicillins	901	12.6
Macrolides	799	11.1
Cephalosporins	561	7.8
Sulfonamides and trimethoprim	445	6.2
Tetracyclines	295	4.1
Amphenicols	109	1.5
Nontyphoidal *Salmonella* infection, 418 cases		
Quinolones	127	30.4
Macrolides	105	25.1
Penicillins	88	21.0
Sulfonamides and trimethoprim	59	14.1
Nitroimidazoles	21	5.0
Cephalosporins	13	3.1
Tetracyclines	4	1.0
Amphenicols	1	0.2
*Campylobacter* infection, 1,165 cases		
Macrolides	826	70.9
Quinolones	243	20.9
Nitroimidazoles	58	5.0
Tetracyclines	12	1.0
Penicillins	10	0.9
Cephalosporins	8	0.7
Sulfonamides and trimethoprim	5	0.4
Amphenicols	3	0.3

*Ten prescriptions for acute gastroenteritis are not shown: 7 for nitrofurantoin, 2 for tobramycin and 1 for methenamine.

**Figure 2:**
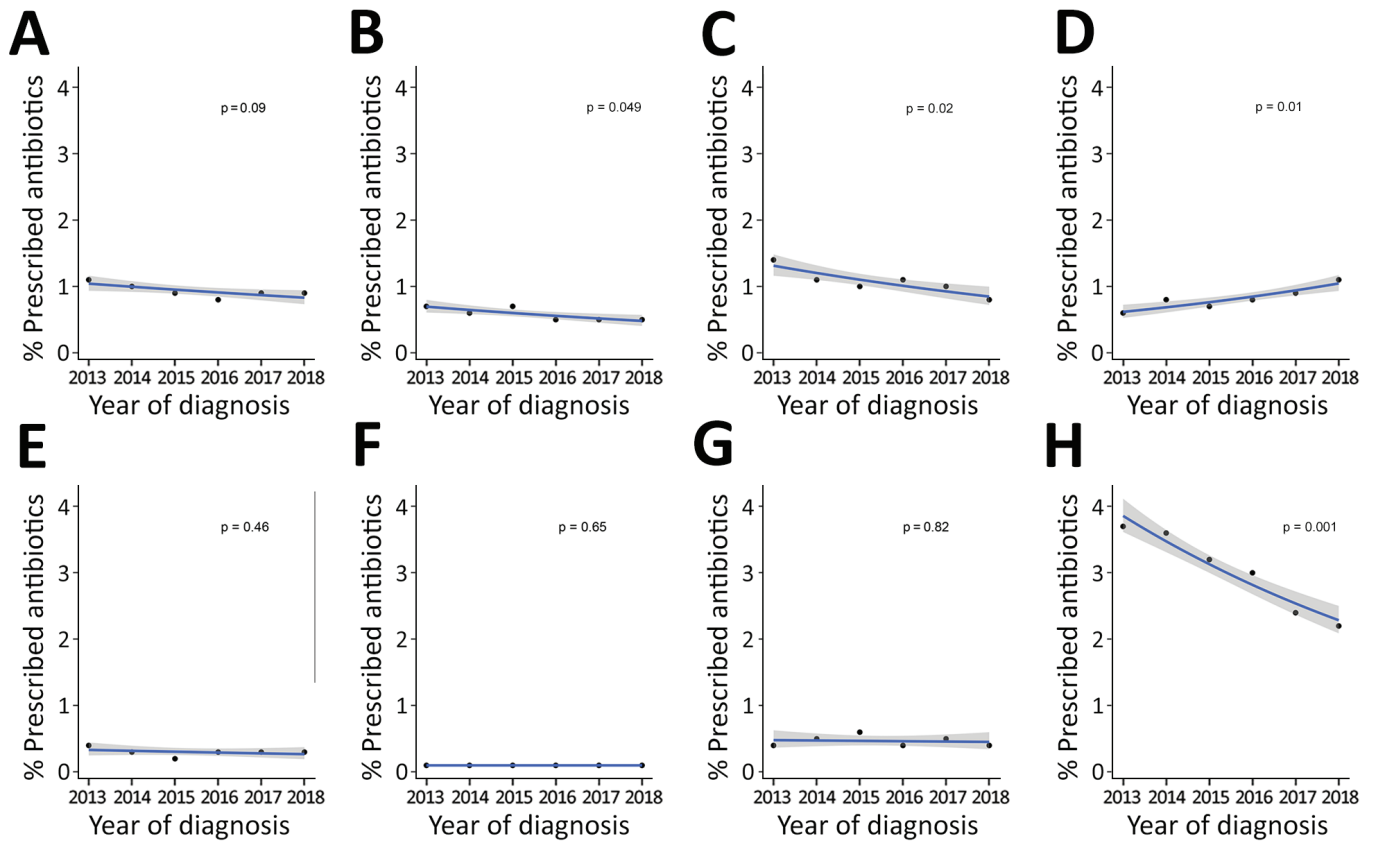
Trend in antimicrobial drug prescriptions for cases of acute gastroenteritis, by year and antimicrobial therapeutic class, Australia, 2013–2018. Ten prescriptions acute gastroenteritis are not shown: 7 for nitrofurantoin, 2 for tobramycin, and 1 for methenamine. A) Penicillins; B) cephalosporins; C) quinolones; D) macrolides; E) tetracyclines; F) amphenicols; G) sulfonamides and trimethoprim; H) nitroimidazoles.

Prescriptions of cephalosporins, quinolones, and nitroimidazoles decreased significantly over the study period (Figure 2). The greatest reduction was for nitroimidazoles (absolute reduction from 3.9% to 2.3%; p = 0.001), followed by quinolones (1.3% to 0.8%; p = 0.02) and cephalosporins (0.7% to 0.5%; p = 0.049). However, prescriptions of macrolides increased significantly (0.6% to 1.0%; p = 0.01).

For the 391 cases of nontyphoidal *Salmonella* infection, a total of 418 prescriptions were written: 1 for 93.1% (364/391) and 2 for 6.9% (27/391). No dominant antimicrobial drugs were prescribed for patients with nontyphoidal *Salmonella*; most commonly prescribed were quinolones (30.4% of total; Table 2). For 1,066 cases of *Campylobacter* infection, 1,165 prescriptions were written: 1 for 91.0% (970/1,066) and >2 (range 2–4) for 9.0% (96/1,066). The predominant antimicrobial drugs prescribed for *Campylobacter* infections were macrolides (70.9% of total; Table 2), of which most were azithromycin (44.4% of total; Appendix Table 4).

## Conclusions

In this large study of patient clinical encounters in general practices in Australia, we found that antimicrobial drugs were prescribed for 6.8% of all cases of acute gastroenteritis but for 35.7% of nontyphoidal *Salmonella* infections and 54.1% of *Campylobacter* infections. Over the 6-year study period, the absolute proportion of cases for which antimicrobial drugs were prescribed for acute gastroenteritis decreased by 2%.

Of the few studies reporting on how often antimicrobial drugs are prescribed for acute gastroenteritis, estimates range from 8.5% of 2,089 cases in a sentinel surveillance sample from primary care in Switzerland in 2014 (*12*) to 65% in a survey of 237 physicians in China in 2012 (*13*). Our results were most similar to the estimates reported from the Switzerland study, which also found that antimicrobial drugs were more likely to be prescribed for older patients and those with fever (*12*).

In Australia, treatment guidelines recommend that empirical prescription of antimicrobial drugs is of no benefit for acute gastroenteritis and is indicated only for patients with manifestations of severe disease, those who are immunocompromised, returned travelers of all ages, or children in whom systemic bacterial infection is suspected (*7*). Our results suggest that general practitioners are more likely to adhere to guidelines and that antimicrobial drugs are more likely to be prescribed for patients who are older, those with underlying conditions, and those with systemic symptoms (e.g., fever). However, the substantial numbers of patients without these indications for whom antimicrobial drugs were still prescribed suggests overuse of antimicrobial drugs for acute gastroenteritis.

Reassuringly, we did find reduced antimicrobial drug prescriptions for acute gastroenteritis during the 6-year study period. This finding is consistent with that of an earlier study that used the same dataset and found an overall reduction in the proportion of patients for whom systemic antimicrobial drugs were prescribed: from 31.7% in 2015 to 26% in 2017 (*14*). This reduction has been attributed to a series of antimicrobial stewardship programs implemented during 2009–2014, which included educational and advertising campaigns aimed at general practitioners and consumers (*15*). Our results suggest that these antimicrobial stewardship programs may have reduced antimicrobial drug prescriptions for acute gastroenteritis.

Given the estimated 1.1 million cases of acute gastroenteritis seen in general practices in Australia annually (*3*), we estimate that nationwide ≈74,000 antimicrobial drugs are prescribed for acute gastroenteritis every year. Because most of these drugs are probably unnecessary, our findings highlight the need for greater antimicrobial stewardship to support management of infectious gastroenteritis in primary care.

Appendix Supplemental methods and results for study of prescribing antimicrobial drugs for acute gastroenteritis, primary care, Australia, 2013–2018.
