# Supplementary material for: Prescribing Antimicrobial Drugs for Acute Gastroenteritis, Primary Care, Australia, 2013–2018
Source: Emerg Infect Dis. 2021 May;27(5):1462–7. doi: 10.3201/eid2705.203692 (PMC8084523; doi:10.3201/eid2705.203692)
Supplement: Appendix — Supplemental methods and results for study of prescribing antimicrobial drugs for acute gastroenteritis, primary care, Australia, 2013–2018. [file 20-3692-Techapp-s1.pdf]

Article DOI: https://doi.org/10.3201/eid2705.203692

Prescribing Antimicrobial Drugs for Acute Gastroenteritis, Primary Care, Australia, 2013–2018

Appendix

Methods

Participants, data sources and definitions

MedicineInsight is an Australian national primary health care database of longitudinal de-identified electronic health records established in 2011. A detailed description of the database has been published previously (*1*). In 2018, MedicineInsight had 662 participating practices, representing approximately 8.2% of all general practices in Australia and 2.3 million regular patients (*1*). The dataset consists of practice and patient information including demographics, diagnosis, encounter reason, observations recorded, pathology requests, and prescription data. While data are anonymised, each patient, site and provider have a unique identifying number which can be used to link all the records held in the database for an individual.

For this study, a simple random sample of 25% of all patients with records between 1st January 2013 and 31st December 2018 was used. To ensure data quality, practices were excluded from analyses if they had low patient volume (less than 100 records in any diagnosis, encounter reason or prescription tables in any study year). From the remaining practices, we extracted clinical encounter records for cases of acute gastroenteritis, non-typhoidal salmonella or campylobacter infection. These were defined based on specific terms in the encounter reason or diagnosis fields (see Appendix Table 5). Multiple encounters with the same case definition within 30 days were counted as the same episode. For each episode that met our case definition, we examined whether there was an antibiotic prescribed on the day of diagnosis (see Appendix Figure 1 for the distribution of antibiotic prescribing date in relation to case diagnosis date) and if there was, we examined the class of antibiotic prescribed and the reason for prescription.

Antibiotics were identified by their medicine active ingredients and categorised based on the Anatomical Therapeutic Chemical Classification System (*2*).

Analysis

For each of acute gastroenteritis, non-typhoidal *Salmonella* and *Campylobacter*, we estimated the proportion of encounters where antibiotics were prescribed on the diagnosis date overall and then by various patient characteristics. These included age at encounter (<10, 10-29, 30-49, 50-64, 65+ years), sex (male, female), Indigenous status (no, yes), concession card holders referring to people with a Pensioner Concession Card, a Commonwealth Seniors Health Card or a Health Care card (no, yes), smoking (never, past, current), fever >38.5 ^{o}C (no, yes, not recorded), stool sample test requested (not recorded, yes), etiology (not recorded, viral, bacterial, parasitic), comorbidity (any medical history of diabetes, arthritis, or chronic kidney disease: no, yes), number of GP visits for clinical encounters in year prior to diagnosis (0-7, 8-14, 15+), and year of diagnosis (2013, 2014, 2015, 2016, 2017, 2018). Practice characteristics included practice remoteness (major city, inner regional, outer regional or remote). To account for multiple episodes in the same patient, generalised estimating equations (GEE) with exchangeable correlation structure was used to estimate characteristics associated with higher likelihood of antibiotic prescribing. Crude and adjusted odds ratios (ORs) were calculated with corresponding 95% confidence intervals (95% CI). A two-sided P value lower than 0.05 was considered statistically significant.

To understand the trends in antibiotic prescriptions over the study period, the proportions of antibiotic prescriptions by the year of diagnosis were then modelled with log-linear regression overall, by age and antimicrobial therapeutic classes. For the calculation of absolute reduction and increase, we used the first minus the last fitted value.

All analyses were performed using R version 3.5.1 (*3*).

Appendix Table 1. Proportion of episodes of acute gastroenteritis prescribed antibiotics overall and according to various characteristics for the children younger than 10 years.

Variable	N Prescribed/N acute gastroenteritis (%)	Adjusted Odds Ratios (95% CI)	P value*
Overall	762/20130 (3.8)		
Sex			
Male	410/10666 (3.8)	Ref	
Female	352/9464 (3.7)	0.97 (0.83, 1.12)	0.64
Aboriginal or Torres Strait Islander			
No	568/14680 (3.9)	Ref	
Yes	42/732 (5.7)	1.51 (1.10, 2.09)	0.01
Unknown	152/4718 (3.2)		
Concession card holder			
No	443/13007 (3.4)	Ref	
Yes	152/3788 (4.0)	1.16 (0.96, 1.41)	0.12
Unknown	167/3335 (5.0)		
Fever (>38.5 ºC)			
No	245/7137 (3.4)	Ref	
Yes	21/277 (7.6)	2.37 (1.51, 3.71)	<0.001
Not recorded	496/12716 (3.9)	1.13 (0.96, 1.32)	0.13
Stool sample test requested			
No	599/17252 (3.5)	Ref	
Yes	163/2878 (5.7)	1.64 (1.36, 1.97)	<0.001
Etiology			
Not recorded	636/14622 (4.3)	Ref	
Viral	71/5387 (1.3)	0.35 (0.27, 0.44)	<0.001
Bacterial	55/119 (46.2)	19.77 (13.46, 29.05)	<0.001
Comorbidity#			
No	759/20057 (3.8)	Ref	
Yes	3/73 (4.1)	1.09 (0.34, 3.46)	0.88
Number of GP visit in last year			
0-7	583/14801 (3.9)	Ref	
8-14	128/3633 (3.5)	0.89 (0.73, 1.09)	0.26
15+	51/1696 (3.0)	0.77 (0.57, 1.03)	0.08
Remoteness of practice			
Major city	501/14991 (3.3)	Ref	
Inner regional	127/3309 (3.8)	1.14 (0.93, 1.40)	0.20
Outer regional or remote	134/1830 (7.3)	2.33 (1.91, 2.84)	<0.001
Year of diagnosis			
2013	131/3064 (4.3)	Ref	
2014	159/3210 (5.0)	1.17 (0.92, 1.48)	0.19
2015	153/3632 (4.2)	0.99 (0.78, 1.26)	0.96
2016	116/3606 (3.2)	0.74 (0.57, 0.96)	0.02
2017	118/3742 (3.2)	0.73 (0.57, 0.94)	0.02
2018	85/2876 (3.0)	0.69 (0.52, 0.92)	0.01

#Comorbidity refers to any medical history of diabetes, arthritis, or chronic kidney disease.
*Adjusted for all the variables listed in the table.

Appendix Table 2. Proportion of episodes of non-typhoidal *Salmonella* prescribed antibiotics overall and according to various characteristics, 2013-2018

Variable	N Prescribed/N non-typhoidal salmonella (%)	Adjusted Odds Ratios (95% CI)	P value*
Overall	391/1096 (35.7)		
Age (years)			
<10	101/296 (34.1)		
10-29	79/208 (38.0)	1.32 (0.89, 1.96)	0.17
30-49	111/266 (41.7)	1.56 (1.08, 2.27)	0.02
50-64	49/160 (30.6)	0.96 (0.59, 1.55)	0.85
65+	51/166 (30.7)	0.95 (0.54, 1.69)	0.87
Sex			
Male	163/483 (33.7)		
Female	228/613 (37.2)	1.18 (0.90, 1.54)	0.23
Aboriginal or Torres Strait Islander			
No	306/841 (36.4)		
Yes	8/23 (34.8)	0.93 (0.42, 2.06)	0.86
Unknown	77/232 (33.2)		
Concession card holder			
No	193/579 (33.3)		
Yes	103/301 (34.2)	1.30 (0.87, 1.95)	0.21
Unknown	95/216 (44.0)		
Fever (>38.5 ^{o}C)			
No	49/97 (50.5)		
Yes	3/6 (50.0)	1.25 (0.27, 5.73)	0.78
Not recorded	339/993 (34.1)	0.45 (0.28, 0.72)	<0.01
Stool sample test requested			
Not recorded	351/989 (35.5)		
Yes	40/107 (37.4)	0.95 (0.60, 1.52)	0.84
Comorbidity#			
No	310/857 (36.2)		
Yes	81/239 (33.9)	1.13 (0.73, 1.75)	0.60
Number of GP visits in last year			
0-7	234/631 (37.1)		
8-14	97/250 (38.8)	1.12 (0.81, 1.55)	0.49
15+	60/215 (27.9)	0.70 (0.47, 1.04)	0.08
Remoteness of practice			
Major city	224/650 (34.5)		
Inner regional	78/267 (29.2)	0.81 (0.57, 1.14)	0.23
Outer regional or remote	89/179 (49.7)	1.96 (1.30, 2.94)	<0.01
Year of diagnosis			
2013	53/126 (42.1)		
2014	82/222 (36.9)	0.73 (0.45, 1.16)	0.18
2015	76/200 (38.0)	0.86 (0.53, 1.38)	0.52
2016	73/224 (32.6)	0.66 (0.41, 1.05)	0.08
2017	64/184 (34.8)	0.74 (0.45, 1.21)	0.23
2018	43/140 (30.7)	0.59 (0.34, 1.02)	0.06

[#]Comorbidity refers to any medical history of diabetes, arthritis, or chronic kidney disease.
*Adjusted for all the variables listed in the table.

Appendix Table 3. Proportion of episodes of *Campylobacter* prescribed antibiotics overall and according to various characteristics, 2013-2018

Variable	N Prescribed/N campylobacter (%)	Adjusted Odds Ratios (95% CI)	P value*
Overall	1066/1969 (54.1)		
Age (years)			
<10	139/271 (51.3)		
10-29	241/446 (54.0)	1.22 (0.89, 1.66)	0.21
30-49	285/497 (57.3)	1.33 (0.98, 1.81)	0.07
50-64	205/362 (56.6)	1.23 (0.87, 1.74)	0.23
65+	196/393 (49.9)	1.00 (0.67, 1.48)	1.00
Sex			
Male	529/1023 (51.7)		
Female	537/946 (56.8)	1.24 (1.03, 1.50)	0.02
Aboriginal or Torres Strait Islander			
No	832/1528 (54.5)		
Yes	18/24 (75.0)	2.35 (0.91, 6.12)	0.08
Unknown	216/417 (51.8)		
Concession card holder			
No	529/1008 (52.5)		
Yes	319/609 (52.4)	0.96 (0.74, 1.24)	0.75
Unknown	218/352 (61.9)		
Fever (>38.5 ^{o}C)			
No	88/139 (63.3)		
Yes	6/6 (100.0)		
Not recorded	972/1824 (53.3)		
Stool sample test requested			
Not recorded	990/1841 (53.8)		
Yes	76/128 (59.4)	1.28 (0.88, 1.87)	0.20
Comorbidity#			
No	789/1465 (53.9)		
Yes	277/504 (55.0)	1.13 (0.86, 1.47)	0.39
Number of GP visits in last year			
0-7	560/1047 (53.5)		
8-14	279/497 (56.1)	1.11 (0.88, 1.39)	0.38
15+	227/425 (53.4)	1.00 (0.77, 1.30)	0.98
Remoteness of practice			
Major city	636/1219 (52.2)		
Inner regional	262/466 (56.2)	1.13 (0.89, 1.44)	0.33
Outer regional or remote	168/284 (59.2)	1.25 (0.93, 1.69)	0.14
Year of diagnosis			
2013	116/208 (55.8)		
2014	174/319 (54.5)	0.90 (0.63, 1.29)	0.56
2015	198/375 (52.8)	0.87 (0.62, 1.23)	0.44
2016	204/357 (57.1)	1.06 (0.75, 1.51)	0.74
2017	170/353 (48.2)	0.77 (0.54, 1.11)	0.16
2018	204/357 (57.1)	1.09 (0.77, 1.56)	0.62

#Comorbidity refers to any medical history of diabetes, arthritis, or chronic kidney disease.
*Adjusted for all the variables except fever listed in the table.

Appendix Table 4. The five most prescribed antibiotics/antimicrobials for episodes of acute gastroenteritis, non-typhoidal salmonella and campylobacter infections.

Variable	Number of prescriptions	Proportion of total prescriptions (%)
Acute gastroenteritis (n=7159)		
Metronidazole	1771	24.7
Tinidazole	1067	14.9
Norfloxacin	602	8.4
Ciprofloxacin	457	6.4
Amoxicillin	452	6.3
Non-typhoidal *Salmonella* (n=418)		
Azithromycin	93	22.2
Ciprofloxacin	91	21.8
Amoxicillin	66	15.8
Norfloxacin	36	8.6
Sulfonamides and trimethoprim	31	7.4
Campylobacter (n=1165)		
Azithromycin	517	44.4
Erythromycin	156	13.4
Norfloxacin	140	12.0
Erythromycin	132	11.3
Ciprofloxacin	103	8.8

Appendix Table 5. Terms used to identify a diagnosis of acute gastroenteritis, non-typhoidal salmonella, and campylobacter.

Variable	Terms for inclusion	Terms for exclusion
Acute gastroenteritis	“gastro”, “gastroenteritis”, “gastro enteritis”, “gastroentritis”, “gastro enterities”, “gastro-enteritis”, “gastroentrist”	"chronic", “likely”, “letter”, “refer”, “referral”, “gastroscopoy”, “upset”, “tiredness”, “or”, “skype”, “gastroschisis”, “gastroenterologist”, “gastro-intestinal”, “protection”, “cancer”, “gastroprotective”, “gastrojejunostomy”, “gastroenterostomy”, “bleeding”, “gastrosleeve”, “gastrostasis”, “muscle”, “torn”, “travel”, “?”, "cramp", "plasty"
Non-typhoidal *Salmonella*	“salmonella”, "salmonellosis"	“typhi”, "paratyphi", “immunization”, “?”, “age”, “post”, “suspicion”, “immunology”, “post”, “review”, “recall”, “trip”, “was”, “urine”, “vaccination”
Campylobacter	“campylobacter”, “notification”	“?”, “not”, “suspected”, “contact”, “likely”, “previous”, “post”, “resolved”

We used the following algorithm to include records of these infections:
1. had any of the inclusion terms in any of the following fields: encounter reason or diagnosis;
2. but was not accompanied by any of the exclusion terms.

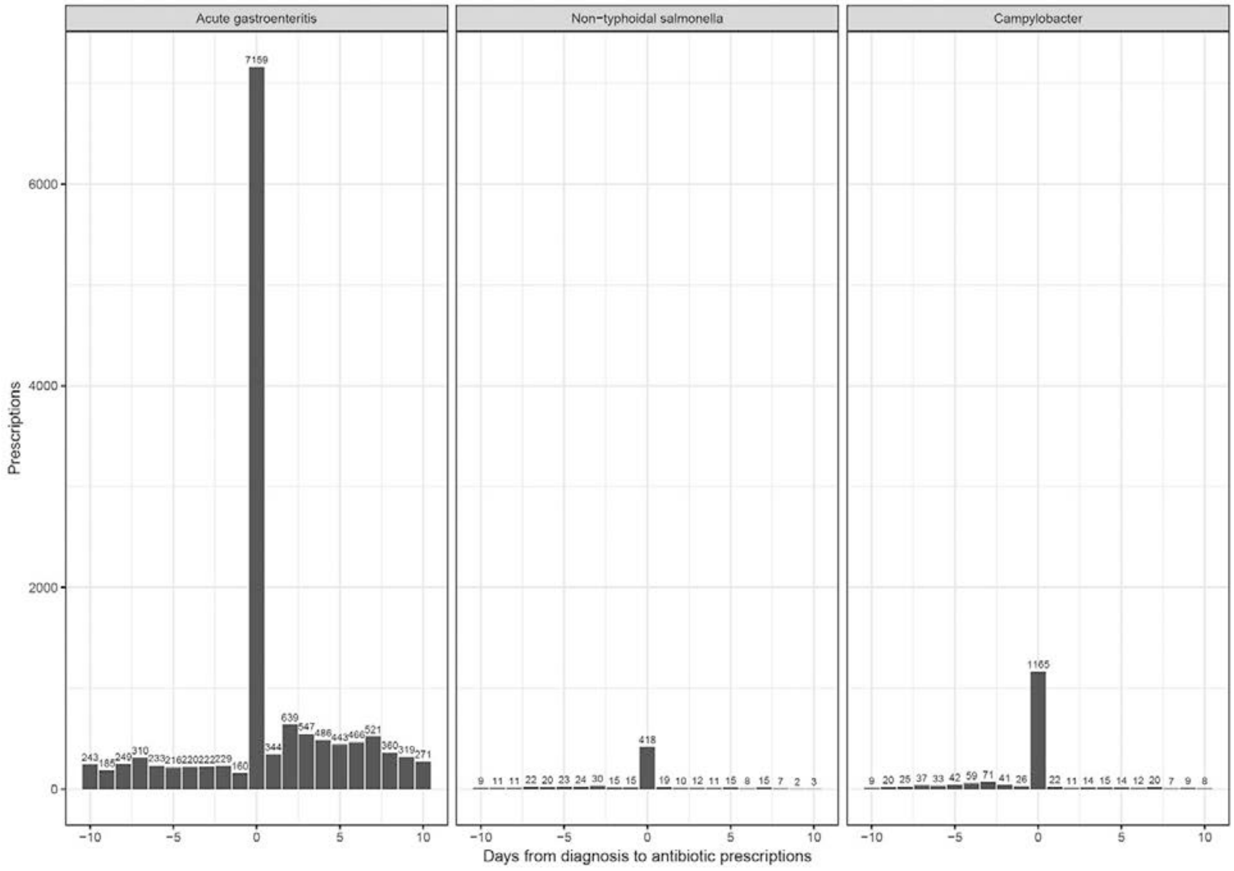

Appendix Figure. Timing of the antibiotics prescription in relation to presentation for acute gastroenteritis, non-typhoidal *Salmonella* and *Campylobacter* infections.

References

1. Busingye D, Gianacas C, Pollack A, Chidwick K, Merrifield A, Norman S, et al. Data Resource Profile: MedicineInsight, an Australian national primary health care database. Int J Epidemiol. 2019 Dec 1;48(6):1741-h.

2. World Health Organization, Norwegian Institute of Public Health. Anatomical therapeutic chemical (ATC) classification system [cited 2020 Mar 28]. https://www.who.int/tools/atc-ddd-toolkit/atc-classification.

3. Team RCR. A language and environment for statistical computing [cited 2020 Mar 28]. https://www.gbif.org/tool/81287/r-a-language-and-environment-for-statistical-computing
